# Effect of a temperature gradient on the behaviour of an endangered Mexican topminnow and an invasive freshwater fish

**DOI:** 10.1038/s41598-022-24755-9

**Published:** 2022-11-29

**Authors:** Sebastian Gomez-Maldonado, Morelia Camacho-Cervantes

**Affiliations:** Instituto de Ciencias del Mar y Limnologia, Universidad Nacional Autonoma de Mexico (UNAM), Ciudad Universitaria, 04510 Mexico City, Mexico

**Keywords:** Behavioural ecology, Freshwater ecology, Invasive species

## Abstract

Climate change and biological invasions are two of the major threats to biodiversity. They could act synergistically to the detriment of natives as non-native species may be more plastic and resilient when facing changing environments. The twoline skiffia (*Skiffia bilineata*) is an endangered Mexican topminnow that cohabits with invasive guppies (*Poecilia reticulata*) in some areas in central Mexico. Guppies have been found to take advantage from associating with the twoline skiffia and are considered partially responsible for the decline of its populations. Refuge use and exploratory behaviours are trade-offs between being safe from the unknown and the opportunity to explore novel areas in search for better resources or to disperse. The aim of this study is to investigate how a change in temperature affects the refuge use and exploratory behaviours for both species. We found that temperature affects the refuge use of twoline skiffias, and the swimming activity of both species. Skiffias explored the rock more than guppies regardless of the temperature scenario. Also, smaller fish spent more time performing exploratory behaviours than bigger ones. Our study is the first to test the effect of temperature on the refuge use and exploratory behaviour of a goodeid species, and our results contribute to the idea that some natives could be more affected by climate change than some invaders.

## Introduction

Freshwater ecosystems are among the most diverse in the world^1,2^, and contribute to both the well-being of humans and the survival of other species^3^. These ecosystems are highly vulnerable to anthropogenic pressures that directly affect the nutrients cycles and community assemblages^4^. Among these pressures, invasive species and climate change are of the most importance^5^. Climate change is causing an increase in average temperatures, but it can also cause a considerable decrease and destabilisation of average temperatures in various areas of the planet^6^. By definition, successful invasive species could be more tolerant to climate change because they tend to be more resilient, while native species tend to be more specific in their tolerance ranges^7–9^.

Climate change has been shown to have a relevant impact on both the spread and distribution of invasive species. Some of these species thrive in new locations due to the modification of temperatures; so, the increase or decrease in temperatures could represent an advantage for them^7,10^. Consequently, climate change together with biological invasions pose a joint threat to the maintenance of biodiversity^11,12^.

Temperature changes have important effects on various biological aspects of aquatic biota^13^; such as reproduction rates^14^, growth rates^15^, their geographical distribution^16^ and in their behaviour^17^. So, there are concerns about the increase in temperatures pushing certain species, mainly natives, to their thermal limits. Especially tropical species, whose environmental temperature remains relatively stable throughout the year^18^.

Risk-taking behaviour is key for animal’ survival, even in a non-predator–prey interaction, as it allows individuals to explore novel areas which in turn leads to the opportunity of locating more and/or better resources^19,20^. A way to measure how keen individuals are to take risks is to assess their use of refugia, as well as the ability to explore new environments and/or unknown objects^21^. The longer animals spent in a refuge, the less time they engage in risk-taking behaviours that could lead them to find food, mating partners, or disperse^22,23^. However, since risk-taking behaviour could also result in negative outcomes, an individual continually decides to engage in it or remain sheltered. Some factors that influence this decision are predation risk, individual’s energy status and habitat variations^24,25^. This is of particular importance when considering temperature changes and ectotherm species, as changes in temperature could modify metabolic rates and in turn its behaviours^26^. For example, it has been, demonstrated that changes in temperatures affect the exploratory tendency and learning ability in a topminnow fish^27^.

Fish have a preferred temperature range, and some have been shown to be able to detect environmental changes of up to 1.5 °C^28^. The native area of our endangered subject species (the twoline skiffia, *Skiffia bilineata*), where guppies (*Poecilia reticulata*) are recognised as invaders, is Central Mexico. Predictions of climate change scenarios for this area are scarce; the closest to our native species’ distribution shows an increase in temperature of 1.4 °C by 2030, 2.2 °C by 2060 and 3.6 °C by 2090^29^. Similarly, for north-eastern Mexico, the mean annual surface temperature is expected to increase 2.5 ± 1.0 °C and more frequent and severe droughts are to happen by the middle of the twenty-first century^6,30^.

Since particular predictions for freshwater bodies are scarce and our focal fish shared common habitats, we tested how a gradient of temperature would influence the refuge use and exploratory behaviours of the native endangered species twoline skiffia compared to the successful invasive guppy in temperatures above and below the ones they experience in the wild. We hypothesise that (1) refuge usage of the invasive guppy will remain similar in different temperature scenarios, while native twoline skiffia will be more affected by temperature changes, and (2) guppies will engage more in exploratory behaviours regardless of the temperature scenario, while native twoline skiffias will be more affected by temperature changes. Our results could contribute to better understand how temperature changes could affect Mexican native freshwater fish compared to an invasive species already recognised as one of their major threats^31^.

## Results

### Time using the rock as refuge

**Figure 1:**
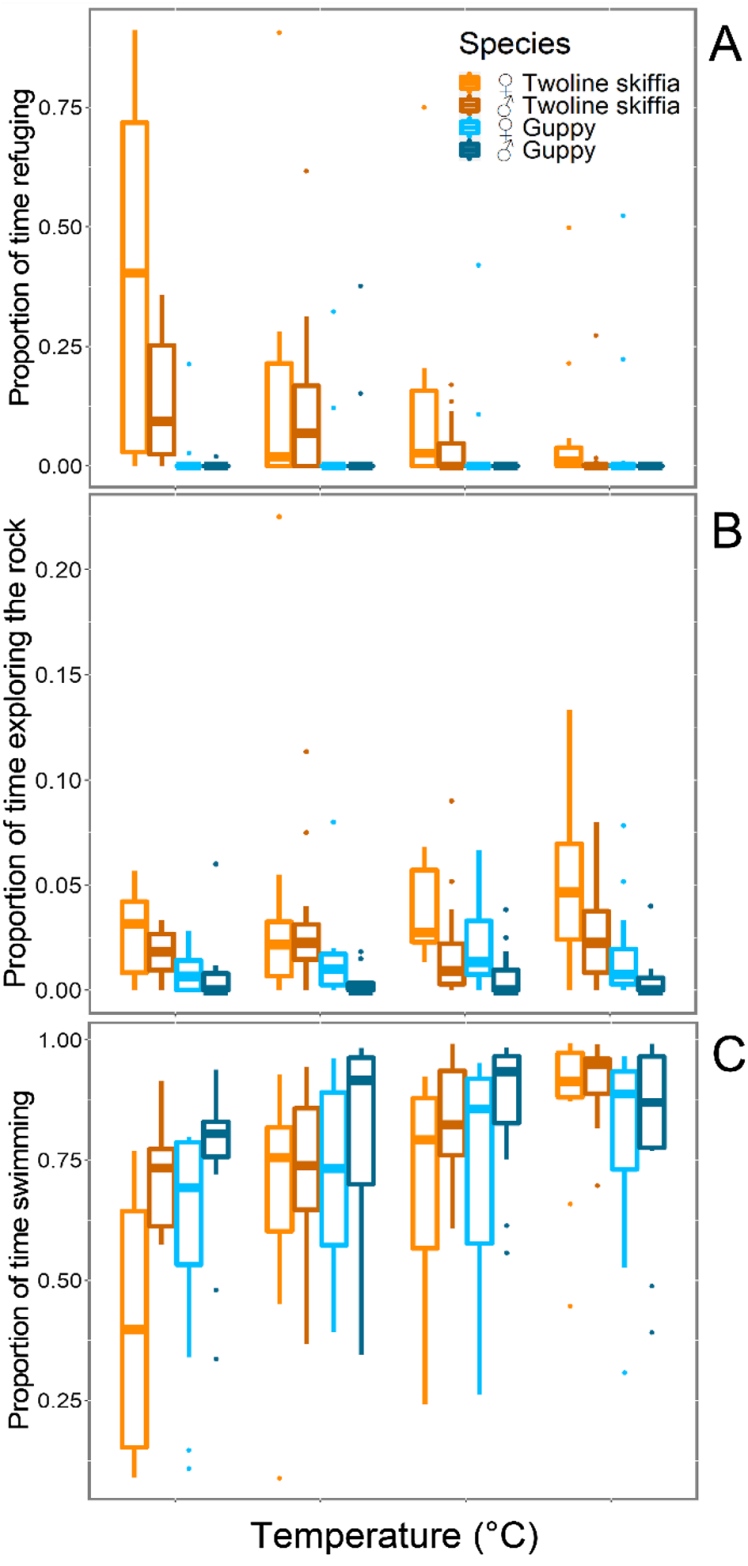
Proportion of the total time observed (600 s) fish: (**A**) used the rock as a refuge, (**B**) spent exploring the rock, and (**C**) spent swimming around the aquarium. Horizontal lines in the bars represent the median and boxes the 50% of the data values.

Temperature had an effect in the refuge usage of both species when analysed together (lme.zig: F_3,192_ = 7.97, p = 0.0001; Fig. 1A). However, species behaved differently (lme.zig: F_1,192_ = 14.79, p = 0.0004; Fig. 1A). As hypothesised, there was an interaction between temperature and species (lme.zig: F_3,192_ = 11.90, p < 0.0001, Fig. 1A), while twoline skiffias decreased their refuge usage as temperature increased (lme.zig: F_3,96_ = 7.26, p = 0.0003, Fig. 1A), guppies showed no change in their behaviour (lme.zig: F_3,96_ = 0.64, p = 0.594, Fig. 1A).

There were differences between sexes (lme.zig: F_1,192_ = 5.70, p = 0.021, Fig. 1A) in the refuge usage and an interaction with temperature (lme.zig: F_3,192_ = 7.14, p = 0.0002, Fig. 1A) of both species when analysed together, however, males and females of the two species behaved differently (lme.zig: F_1,192_ = 3.96, p = 0.053, Fig. 1A). Female twoline skiffias used the rock as a refuge more than males (lme.zig: F_1,96_ = 3.98, p = 0.059, Fig. 1A) and twoline skiffia, as species, used less the refuge when temperature increased (lme.zig: F_3,96_ = 2.82, p = 0.046, Fig. 1A), whereas guppies showed no differences between sexes (lme.zig: F_1,96_ = 1.41, p = 0.249; Fig. 1A), and there was no interaction with temperature (lme.zig: F_1,96_ = 0.93, p = 0.432; Fig. 1A).

**Figure 2:**
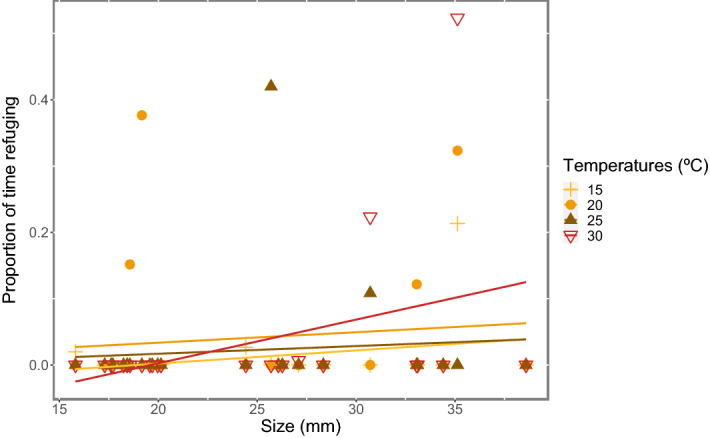
Proportion of the total time observed (600 s) guppies of different sizes used the rock as refuge in the temperature gradient.

Size had no effect in the time spent using the refuge (lme.zig: F_1,192_ = 2.16, p = 0.15) neither for twoline skiffias (lme.zig: F_1,96_ = 1.13, p = 0.301) nor for guppies (lme.zig: F_1,96_ = 0.99, p = 0.332). However, for guppies, there was an interaction between temperature and size (lme.zig: F_3,96_ = 2.76, p = 0.049, Fig. 2), suggesting that at 30 °C they hardly used the refuge, and bigger fish used the refuge more than smaller fish.

### Time exploring the rock

We found that temperature did not had an effect in the time both fish explored the rock when analysed together (lme: F_3,192_ = 1.09, p = 0.345, Fig. 1B). However, species behaved differently (lme: F_1,96_ = 23.41, p < 0.0001, Fig. 1B), twoline skiffias interacted more with the rock than guppies. This was confirmed when species were analysed separately, temperature did not affect the behaviour of twoline skiffias (lme: F_3,96_ = 1.03, p = 0.387, Fig. 1B) or guppies (lme: F_3,96_ = 0.88, p = 0.455, Fig. 1B) and there was no interaction between the temperatures and species (lme: F_3,192_ = 0.90, p = 0.445, Fig. 1B).

In the analysis including both species, sex was not a significant variable (lme: F_1,192_ = 1.92, p = 0.173, Fig. 1B) for the exploring behaviour and there were no interactions between temperature (lme: F_3,192_ = 2.37, p = 0.073, Fig. 1B) and species (lme: F_3,192_ = 1.20, p = 0.280, Fig. 1B). Still, in the analysis for each species we found that female and male guppies behaved differently (lme: F_1,96_ = 6.60, p = 0.018, Fig. 1B), females explored more the rock than males. When twoline skiffias were analysed solely, we did not find any significant interactions (lme: F_1,96_ > 0.30, p > 0.14, Fig. 1B).

**Figure 3:**
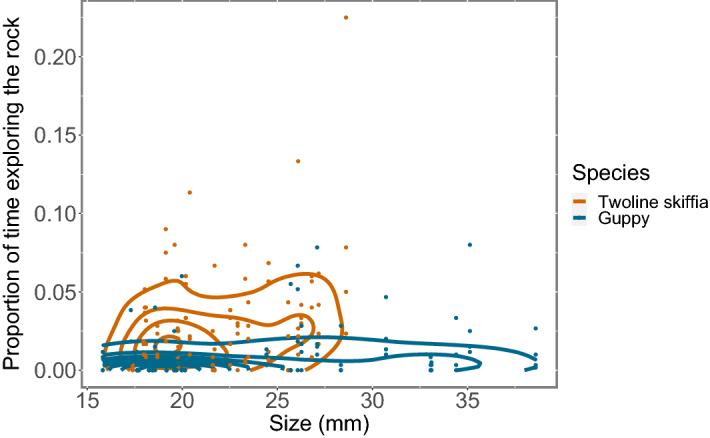
Proportion of the total time observed (600 s) fish of different sizes spent exploring the rock. Lines represent the areas where the density of data is higher.

Size had an effect in the time exploring the rock (lme: F_1,192_ = 6.91, p = 0.012, Fig. 3) when species were analysed together, but there was no interaction with temperatures (lme: F_3,192_ = 0.42, p = 0.74, Fig. 3). We found that the interaction between species and size was close to be significant (lme: F_1,192_ = 3.62, p = 0.064, Fig. 3), implying that possibly smaller fish spent more time exploring the rock than bigger fish. However, when analysed separately, we did not find an effect of size in the exploring behaviour neither for twoline skiffias (lme: F_1,96_ = 2.99, p = 0.099, Fig. 3) nor for guppies (lme: F_1,96_ = 0.33, p = 0.569, Fig. 3).

### Time spent swimming

Temperature had an effect in the time spent swimming for both species when analysed together (lme: F_3,192_ = 23.48, p < 0.0001, Fig. 1C), and species behaved similarly (lme: F_3,192_ = 0.61, p = 0.440, Fig. 1C). We found a significant interaction between temperature and species (lme: F_3,192_ = 4.03, p = 0.009, Fig. 1C), results of analysis for each species showed that twoline skiffias spent more time swimming around the aquarium when temperature increased (lme: F_1,96_ = 24.74, p < 0.0001, Fig. 1C), and guppies behaved similarly but the effect seemed to be weaker (lme: F_1,96_ = 4.68, p = 0.005, Fig. 1C).

We found no differences between sexes (lme: F_1,192_ = 3.33, p = 0.075, Fig. 1C) in the time spent swimming for both species when considered together. However, when analysed separately, males of twoline skiffias spent more time swimming around the aquarium than females (lme: F_1,96_ = 5.22, p = 0.033, Fig. 1C), while guppy males and females spent a similar proportion of time swimming (lme: F_1,96_ = 2.90, p = 0.103, Fig. 1C). There was no interaction with temperature (lme: F_3,96_ = 1.49, p = 0.219, Fig. 1C) neither for twoline skiffias (lme: F_3,96_ = 2.35, p = 0.081, Fig. 1C) nor for guppies (lme: F_3,96_ = 0.56, p = 0.640, Fig. 1C).

**Figure 4:**
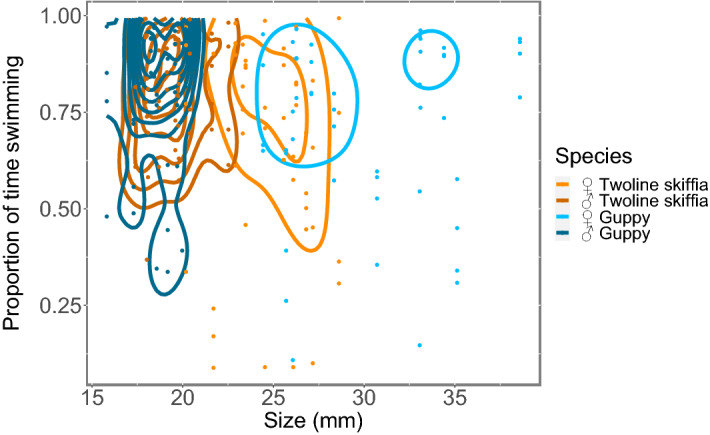
Proportion of the total time observed (600 s) fish of different sizes spent swimming around the aquarium. Lines represent the areas where data density is higher.

Size had an effect in the time spent swimming around the aquarium (lme: F_1,192_ = 4.19, p = 0.047, Fig. 4) when species were analysed together, suggesting that smaller fish spent more time swimming than bigger ones. But when analysed separately, this variable had no effect in the time spent swimming neither for twoline skiffias (lme: F_1,96_ = 0.11, p = 0.748, Fig. 4) nor for guppies (lme: F_1,96_ = 0.23, p = 0.637, Fig. 4).

## Discussion

Temperature affects the refuge usage of twoline skiffias, and the swimming activity of both species, but not the time guppies or skiffias spent exploring the rock. Twoline skiffias used the rock as a refuge more at lower temperatures and more than invasive guppies, which could be explained by a decrease in their metabolic rate^32^. Invasive guppies almost never used the rock as a refuge, particularly at 30 °C, and had no change in their refuge usage behaviour and time spent exploring the rock with temperature changes. The swimming activity of both species were affected by the temperature gradient, they spent more time swimming around the aquarium when temperature increased, but for the guppy the effect seemed to be weaker than for the native twoline skiffia. The thermal tolerance of guppies has been tested to be up to 39.6 °C^33^, but twoline skiffias in our pilot experiment displayed consistent stress behaviour at 32 °C. This supports our results by adding to the idea that invasive species are more tolerant to temperature changes and contributes to explain how a decrease or increase in temperature affects fish behaviour.

Females and males of both species behaved differently in their refuge usage and exploratory behaviours. Regarding the refuge usage, the interaction between females and males for both species was close to be significant (p = 0.053), suggesting that female twoline skiffias spent more time using the rock as a refuge than males at lower temperatures while there was no effect for guppies. Female guppies explored the rock more than males, but temperature had no effect. Both species (females and males) swam more around the aquarium as the temperature increased. Ectothermic males and females do not usually differ significantly in their ability to acclimate to their thermal limits and preferences^34^, but the effects of temperature for females of twoline skiffia and guppies in our experiment were higher than for males. The differences in our results cannot be explained by different sizes between males and females, which has been found to enable females to be more plastic in their responses than males^34–36^. We then hypothesise that the differences between females and males are due to their differences in ecological requirements. At least for guppies, while males spend more time looking for mating partners females spent more time in social and foraging activities^51^. However, more research, including more than one fish and one species being observed at the same time, needs to be done to conclude something on the differential effect of temperature for females and males of our subject species. Indeed, social interaction between natives and invaders was found to be significant for the native Iberian toothcarp (*Aphanius iberius*), where Magellan & Garcia-Berthou found females used more an artificial refuge than males when alone but that changed when interacting with the invasive mosquitofish (*Gambusia holbrooki*), males increased their refuge usage significantly due to competition [Magellan, K. & García-Berthou, E. Experimental evidence for the use of artificial refugia to mitigate the impacts of invasive Gambusia holbrooki on an endangered fish. Biol. Invasions 18, 873–882 (2016)].^27^

Despite of our efforts to observe fish roughly the same size, we found a size gradient and, in general, smaller twoline skiffias and guppies spent more time exploring the rock and swimming around the aquarium than bigger ones. However, bigger guppies used more the rock as a refuge than smaller guppies. In an experiment testing another invasive poecilid, they found that bigger invasive porthole livebearers (*Poeciliopsis gracilis*) were bolder than smaller ones^37^. This could suggest that bigger non-native poeciliids might have advantages when facing novel environments, and, according to our results, when facing changing temperatures.

Changes in water temperature modify the pH and dissolved oxygen levels in the water, and together the aquatic habitat of fish^38^. We acknowledge that in our experiment, we did not measure or control these specific variables along the temperature gradient. An increase in water temperature leads to a decrease in dissolved oxygen (DO). In turn, the species’ exploratory behaviours, specially, their swimming activity might be influenced by DO or a combination of DO and water temperature^39^. The relationship between water temperature and pH has the same effect, for example, it has been reported that pH changes modified the avoidance behaviour as well as the swimming activity of the Japanese fat minnow (*Phoxinus lagowskii*)^40^. Even so, we consider our experiment to be sound since all the fish observed in our study experienced the same experimental and stock conditions, we are then confident that the differences we found are valuable to understand how temperature modifies the refuge usage and exploratory behaviours in our tested fish. Nevertheless, since these variables are of importance, we consider further research should be carried out to explore their particular effects on topminnows behaviours, as in wild conditions these do not remain constant between sites as temperature changes.

In our experiment, the native twoline skiffia spent more time swimming around the aquarium than the invasive guppy, while temperature increased, which supports our results that native species used the rock as a refuge more when temperate decreased. This could be explained since invasive species tend to be more resilient to environmental changes than natives^7–9^. An example is that of invasive guppies in Germany, where they were also found to be very resilient to temperature changes and their boldness behaviour was not affected by them^41^. In contrast, both male and female invasive Australian guppies were found swimming less and engaging less in mating behaviour at 18 °C^42^. To the best of our knowledge this is the first study investigating goodeid’s behaviour under different temperature scenarios. In our experiment guppies showed no changes in their refuge use behaviour, as well in the time spent exploring the rock but the increase in their time swimming around the aquarium could represent an advantage when competing with natives. In a larger scale study, a cousin invasive non-native poeciliid species have been found to take advantage of temperature changes in the environment. In 2015, in the Zempoala lagoons in Mexico, a native species of goodeid (*Girardinichthys multiradiatus*) was displaced by the invasive twospot livebearer (*Pseudoxiphophorus bimaculatus*) when the temperature in the lagoons increased^43^.

Studies carried out with other species have found non-natives are more likely to explore than natives; for example, invasive toads approach unknown areas more than native toads^44^. Since risk-taking behaviour is a characteristic linked with dispersion^20,22^. The fact that guppies in our experiment were not affected by temperature changes and increased one of their exploratory behaviours, indicates that likelihood of them dispersing further as temperature in the world changes remains. Invasive guppies have been found to be faster to engage in risk-taking behaviour, and faster when accompanied by other guppies, other non-native poeciliids and even other native similar fish^45,46^. Global warming can affect through direct impacts the diversity and abundance of native species, as well as indirectly by benefiting non-native species^10^. Since guppies are social and have been found to take advantage of socialising with natives^47^, to better predict how climate change could affect poeciliids invasion, the following step would be researching how native and non-native fish behave when interacting with each other under different temperature scenarios, which would be also relevant to test competition for resources. Still, our results contribute to the idea that when facing climate change scenarios, native topminnows would be under higher threat than non-native ones.

## Methods

### Subject species

The twoline skiffia is endemic to the Lerma river basin^48,49^. It is a species that prefers water moderately hard and alkaline, with temperatures between 18 and 24 °C^49^. Since the early 90 s, according to the IUCN Red List this species is categorised as endangered^50^. The guppy (*Poecilia reticulata*) is a freshwater invasive fish originally from Trinidad and Tobago, Guyana, Venezuela and Surinam^51^ and currently is distributed in all continents except Antarctica^52^. Guppies prefer environments with temperatures between 20 and 24 °C, although they could tolerate different environmental conditions such as temperature and salinity oscillation^51,53–55^.

### Experimental design

**Figure 5:**
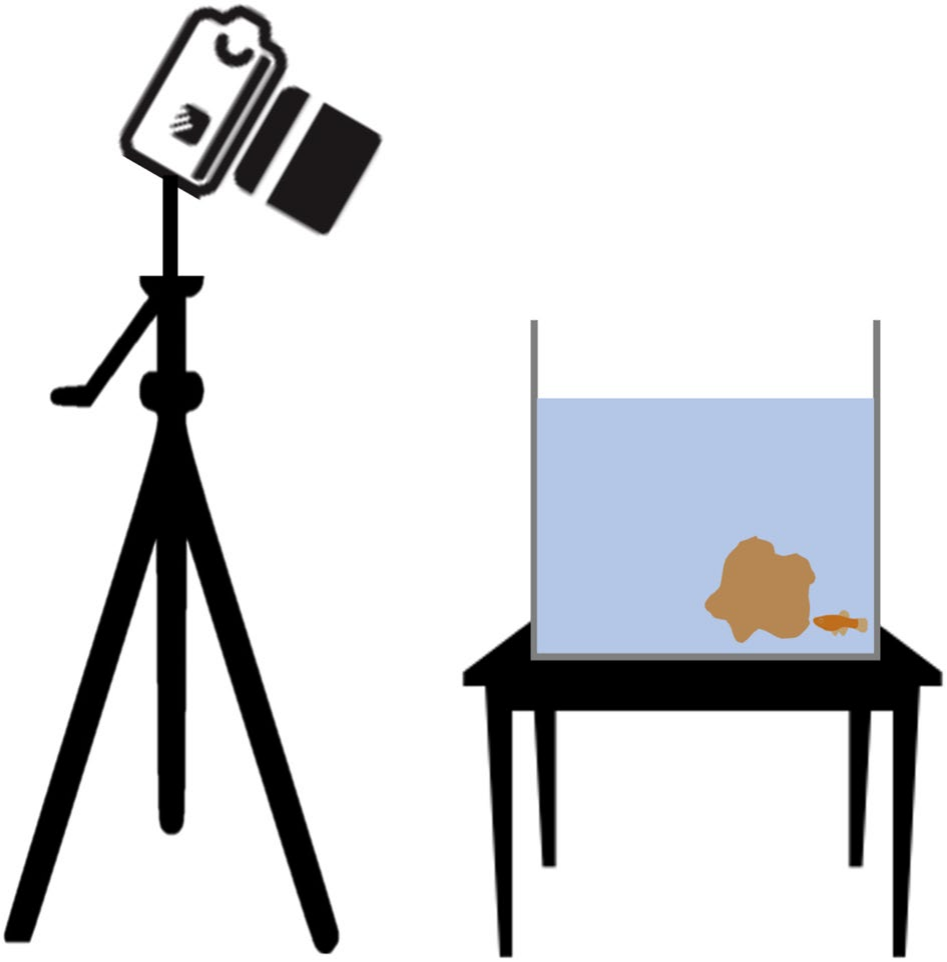
Fish were observed in a 10 L tank that contained only a rock with a diameter of 6.93 cm on its widest. The rock used was beige, similar to twoline skiffias’ and guppies’ predominant colour. Recordings were made with a camera placed at 30° from the top of the tank in order to see the fish at all times.

Experiments were carried out in the Institute of Marine Sciences and Limnology (IMC&L) at UNAM from October 2021 to January 2022. Guppies and twoline skiffias used in this experiment were part of the Invasive Species Ecology Laboratory aquarium experimental collection, located in the IMC&L. Twoline skiffias were originally collected from outdoor ponds in the Institute of Ecology (19° 18′ 44″ N 99° 11′ 46″ W) in 2018, and guppies from Mixquiahuala in the Tula River (20° 30′ 25″ N, 99° 14′ 44″ W) in 2018. Fish in the aquarium are kept in stock tanks (40 L) filled with tap aged water, with gravel at the bottom, a water pump and plastic plants. Photoperiod in the aquarium is 12L:12D. Temperature in the aquarium is kept at 22 °C (± 1 °C), controlled with a MIDEA^®^ air conditioning device. For the purposes of this experiment, the temperature was lowered one degree every day until reaching 15 °C, fish were kept at this temperature for two days and then the experiment started. Experimental fish were kept in 40 L tanks, set up as described above plus a heater (Termojet Ecothermal 50 W Lomas). Fish were separated by species, and only four females and four males were kept in each tank. Observation tank (25 × 20 × 15 cm) was made of transparent glass and included only a rock that fish could use as a refuge (Fig. 5). All fish were kept at the same conditions at all times. Observation tank was filled with water previously set at the experimental temperature. Fish were fed with commercial food flakes (Biomaa^®^) daily, and before being observed.

Fish behaviour was observed in a temperature gradient from 15 to 30 °C, with an interval of 5 °C, we selected these temperatures based on their optimal thermal range and because the only previous study on the effect of temperatures in a goodeid species showed that *Girardinichthys multiradiatus* behaved differently at a minimum change of 4 °C^43^. We stopped the upper thermal range at 30 °C as, in our pilot trials, twoline skiffias showed stress symptoms (e.g., folded fins and odd swimming patterns) when reaching 32 °C. We did not test guppies’ behaviour at higher temperatures since their thermal limits was already explored and was found to be at 39.6 °C^33^. We did not continue the experiment at higher temperatures for guppies as we wanted observations of twoline skiffias and guppies to be equal. Moreover, the model scenarios of temperature changes in an close by area where our species share habitat predict a temperature increase of 3.6 °C^29^, which is almost coincident with the highest temperature tested (30 °C). Temperature was increased one degree a day, followed by 2 days at the experimental temperature and on the third day observations were recorded. Recordings were made with a camera placed at 30° from the top of the tank in order to see the fish at all times (Fig. 5). The rock was placed closer to a corner of the tank and fish were gently released in the opposite corner of the aquarium where the rock was. The rock was randomly alternated between the rear left or right, five cm away from the back glass. Observations lasted 10 min and were recorded using a Nikon D5600 camera with a lens AF-S DX NIKKOR 18–55 mm f/3.5–5.6G VR (set at 18 mm for all observations). For both species we tested males and females as, at least for guppies, male’s behaviour is heavily sexually motivated^50^. Our experiment had a repeated measurement design, we observed individually 48 fish at each of the four temperature scenarios: 12 female twoline skiffias, 12 male twoline skiffias, 12 female guppies, and 12 male guppies. We measured (1) the total time each fish spent using the rock as a refuge, which was when fish approached the rock and remained still right next to it; (2) the total time fish spent exploring the rock, defined as the time fish were facing the rock actively looking at it or seemingly trying to get food from it; and (3) the total time each fish spent swimming around the aquarium without approaching or interacting with the rock. All fish used in this experiment were selected to be roughly the same size. Still, all fish were photographed and measured using the JWatcher^®^ software^56^: average standard body length of 22.06 cm (SD = 3.37 cm) for twoline skiffias and average standard body length of 24.38 cm (SD = 6.8 cm) for guppies. Size was included in the analysis to account for any possible effect.

### Statistical methods

We performed a zero inflated gaussian mixed model (lme.zig function from the R package NBZIMM^57^) to test for the effect of temperature within species in the refuge use behaviour, since this variable was zero-inflated, continuous and derived from a repeated measures experimental design. The variable exploring the rock and swimming were not zero-inflated, so we performed the generic function ‘lme’ (form the R package nlme^58^) to fit a linear mixed effects model to test the effect of temperature in the swimming and exploring behaviour. We tested for normality in our behaviours and since none of them were normally distributed, we Arcsine transformed them for its distribution to approach normality^59^.

For the three registered behaviours we first performed a global analysis using our Arcsine transformed data, which were obtained by expressing them as a proportion of the total time they were observed (600 s). The global analysis included temperature, species, sex and size as explanatory variables. Then, if we found species was significant or was included in a significant interaction, we performed separate analyses to find differences between species including females’ and males’ behaviours and size in the different temperature scenarios within each species. All analyses were carried out in the statistical software R^60^.

### Ethics approval

Experiments for the purposes of this article were conducted at the Universidad Nacional Autonoma de Mexico in Mexico City using fish of two species (Skiffia bilineata, 24 individuals, and *Poecilia reticulata*, 24 individuals) that were already part of research collections, at the Institute of Ecology and the Institute of Marine Sciences and Limnology. The experimental design involved behavioural observations in glass aquarium tanks, which did not include any surgery, anaesthesia, or other invasive procedure that could have produce distress in the fish. Mortality was zero and once the experiments were completed, fish were returned to the aquariums to be kept for future research projects. All methods above were revised and approved by the *Subcomité de Bioética de la Comisión de Ética Académica y Responsabilidad Científica de la Facultad de Ciencias* (Bioethics Subcommittee of the Academic Ethics and Scientific Responsibility Commission of the Faculty of Sciences), UNAM with the folio: PI_2022_01_22; and are in accordance with the Guide for the Care and Use of Laboratory Animals^61^ and ARRIVE guidelines^56^. Fish were transported to the laboratory following the Official Mexican Norm NOM-051-ZOO-1995 for humanitarian treatment in the mobilisation of animals. Laboratory protocols followed all guidelines provided by the Mexican Official Norm NOM-062-ZOO-1999 for the use and maintenance of vertebrates for research purposes.

## Supplementary Information

Supplementary Information.

## Data Availability

Data will be available as supplementary material.
